# Responses of *Piscidia carthagenensis* to Native and Non-Native AMF Inoculation Across Successional Soils from a Xerophytic Shrubland

**DOI:** 10.3390/plants15213386

**Published:** 2026-11-01

**Authors:** Carolina Kalinhoff, Alicia Cáceres, Rosa Urich, Victor Romero

**Affiliations:** 1 Departamento de Ciencias Biológicas y Agropecuarias, Universidad Técnica Particular de Loja, Loja 1101608, Ecuador; 2 Instituto de Biología Experimental, Universidad Central de Venezuela, Caracas 1041-A, Venezuela; alicia2001@gmail.com (A.C.); rosaurich@gmail.com (R.U.); 3 Departamento de Biología, Universidad Simón Bolívar, Caracas 1080-A, Venezuela; vpromero@usb.ve

**Keywords:** AMF inoculum origin, arbuscular mycorrhiza, mycorrhizal inoculation, native AMF consortia, plant–fungus interactions, secondary succession, xerophytic shrubland

## Abstract

Arbuscular mycorrhizal fungi (AMF) can enhance plant establishment in degraded drylands, but inoculation outcomes may depend on inoculum identity and recipient soil conditions. We evaluated the response of *Piscidia carthagenensis* to three native AMF consortia from contrasting successional stages of a xerophytic shrubland and to a non-native *Acaulospora lacunosa* inoculum. Plants were grown in soils representing reference shrubland, early-seral, and late-seral stages. An uninoculated treatment was included as a control. After 112 days, plant growth, nodulation, and AM root colonization were assessed. The magnitude and pattern of plant responses to inoculation varied among the three recipient soils. Native consortia generally produced more consistent positive effects on growth and nodulation than the non-native inoculum, particularly in seral soils. Total root colonization exceeded 90% in inoculated plants grown in early- and late-seral soils, yet plant responses still differed among inocula. Intraradical structures differentiated among inocula more clearly than total root colonization. Home inoculations showed trait-specific advantages over Away inoculations and more consistently outperformed the tested non-native inoculum. These findings suggest that the native AMF consortia evaluated here are promising candidate inocula for the restoration of degraded dryland ecosystems.

## 1. Introduction

Dryland ecosystems, which cover approximately 40% of the Earth’s terrestrial surface, are among the biomes most vulnerable to anthropogenic disturbance and land-use change [1,2]. Human activities, including extractive practices, agricultural expansion, and infrastructure development, can disrupt ecosystem functioning by degrading soil structure and fertility, altering vegetation composition, and limiting the natural regeneration of native plant communities [3]. Restoring these ecosystems, therefore, requires not only the reestablishment of aboveground vegetation but also the recovery of belowground ecological processes and plant–soil interactions that sustain ecosystem resilience [4,5]. Among the biological components driving these interactions, arbuscular mycorrhizal fungi (AMF) play a central role by forming mutualistic associations with the roots of most terrestrial plants. This widespread symbiosis enhances nutrient acquisition, seedling establishment, and tolerance to environmental stress [6]. Moreover, genomic and ecological assessments have revealed that AMF networks are integral components of terrestrial ecosystems and contribute to processes such as nutrient and carbon cycling [7]. These functions are particularly relevant in dryland environments, where plants face persistent water and nutrient limitations and depend strongly on belowground microbial interactions to maintain growth and persistence [8].

AMF inoculation represents a promising strategy to enhance plant establishment during ecological restoration, particularly in arid and semiarid ecosystems [9,10]. Inoculum source can strongly influence restoration outcomes, and locally derived AMF inocula may in some systems outperform commercial inocula [11,12]. Native or environmentally selected AMF inocula can also enhance nutrient acquisition, resource-use efficiency, and plant resilience under stressful conditions, further supporting their potential value for ecological restoration in degraded drylands [10,13]. Beyond individual plant responses, inoculation with native AMF from reference ecosystems can contribute to vegetation recovery by increasing floristic quality and native plant diversity while reducing the abundance of non-native plants in degraded systems [14].

Plant responses to AMF inoculation are inherently context-dependent and may vary with both inoculum identity and recipient soil conditions. Herrera-Peraza et al. [15] showed that the response induced by different AMF strains depends strongly on the properties of the soil into which they are introduced, highlighting soil–inoculum compatibility as an important determinant of inoculation success. Geographic provenance may provide an additional source of variation. Rúa et al. [16], in a meta-analysis of plant–soil–AMF interactions, found that plant responses to mycorrhizal inoculation depended on the geographic origin of both the soil and the symbiotic partners, providing evidence that local adaptation may contribute to variation in symbiotic outcomes. Together, these findings suggest that both recipient soil conditions and inoculum provenance should be considered when evaluating the performance of AMF inocula for ecological restoration.

Although environmental context and soil–AMF compatibility are increasingly recognized as important determinants of inoculation success, it remains unclear whether native AMF consortia originating from different stages of secondary succession differ in their effects on plant performance across successional soils. Ecosystems undergoing secondary succession after severe disturbance provide a valuable setting in which to investigate this question because vegetation recovery is accompanied by changes in soil properties, plant communities, and belowground microbial assemblages. The Macanao Peninsula (Margarita Island, Venezuela) provides such a system. Over recent decades, sand extraction from ephemeral streambeds has profoundly altered xerophytic vegetation by removing native plant cover, stripping the upper soil horizon, and disrupting soil structure, thereby creating a mosaic of sites representing different stages of ecological recovery [17,18]. Previous work in the same study area showed that AMF persisted after disturbance despite changes in vegetation and soil conditions and that AMF assemblages differed between restored and non-restored areas [18]. Long-term revegetation with *Piscidia carthagenensis* also promoted the recovery of several soil chemical, biochemical, and microbiological properties [19]. In the same region, Cáceres-Mago et al. [20] found species-specific growth and physiological responses of native woody plants to enrichment with a locally derived AMF inoculum, further supporting the potential of native AMF for restoration. However, whether native AMF consortia originating from different successional stages differ in their effects when applied to recipient soils representing the same or different stages remains unresolved.

To address this knowledge gap, we used *Piscidia carthagenensis* Jacq., a characteristic leguminous tree of Neotropical seasonally dry forests, to evaluate its responses to AMF inoculation in recipient soils representing three contrasting stages of secondary succession. Three native AMF consortia originating from different successional stages of the tropical xerophytic shrubland were compared with a nono-native *Acaulospora lacunosa* inoculum to examine variation in plant growth, nodulation, and mycorrhizal establishment in relation to inoculum identity and provenance across recipient soils. We tested two specific hypotheses: (1) native AMF consortia would promote greater plant performance than the non-native inoculum; and (2) matching the origin of a native AMF consortium with the successional stage of the recipient soil would enhance plant performance relative to non-matching inoculations. To test these predictions, we addressed the following questions: (1) Does AMF inoculation enhance plant performance in soils representing different stages of secondary succession? (2) Does the origin of native AMF consortia influence plant performance and mycorrhizal establishment when applied to soils corresponding to the same or different successional stages?

## 2. Results

### 2.1. Soils and AMF-Inoculum Description

Soil physicochemical properties differed significantly among the three successional stages (Table 1). Total phosphorus (P), available P, and total nitrogen (N) declined progressively from the reference shrubland to the early-seral stage, with intermediate values in late-seral soils. Organic matter (OM) was similar between shrubland and late-seral soils but was significantly lower in early-seral soils. Likewise, soil pH did not differ between shrubland and late-seral soils but was significantly higher in early-seral soils. Soil texture also varied across the successional gradient, with shrubland soil classified as sandy loam and both seral soils as loamy sand.

Trap cultures yielded 20 AMF morphotypes that differed in composition among the three inoculum sources (Table 2). Although *Claroideoglomus* cf. *etunicatum* was abundant across all inocula, the shrubland-derived inoculum exhibited the greatest morphotype richness and included several morphotypes not detected in the seral inocula, including members of the Gigasporaceae. In contrast, late-seral and early-seral inocula were characterized by fewer morphotypes and were dominated by *Claroideoglomus* cf. *etunicatum*, *Funneliformis geosporum*, *Entrophospora infrequens*, and *Glomus* sp. 1. Thus, the trap-cultured AMF assemblages differed in morphotype composition among inoculum sources.

### 2.2. Plant Growth Responses to AMF Inoculation

**Figure 1:**
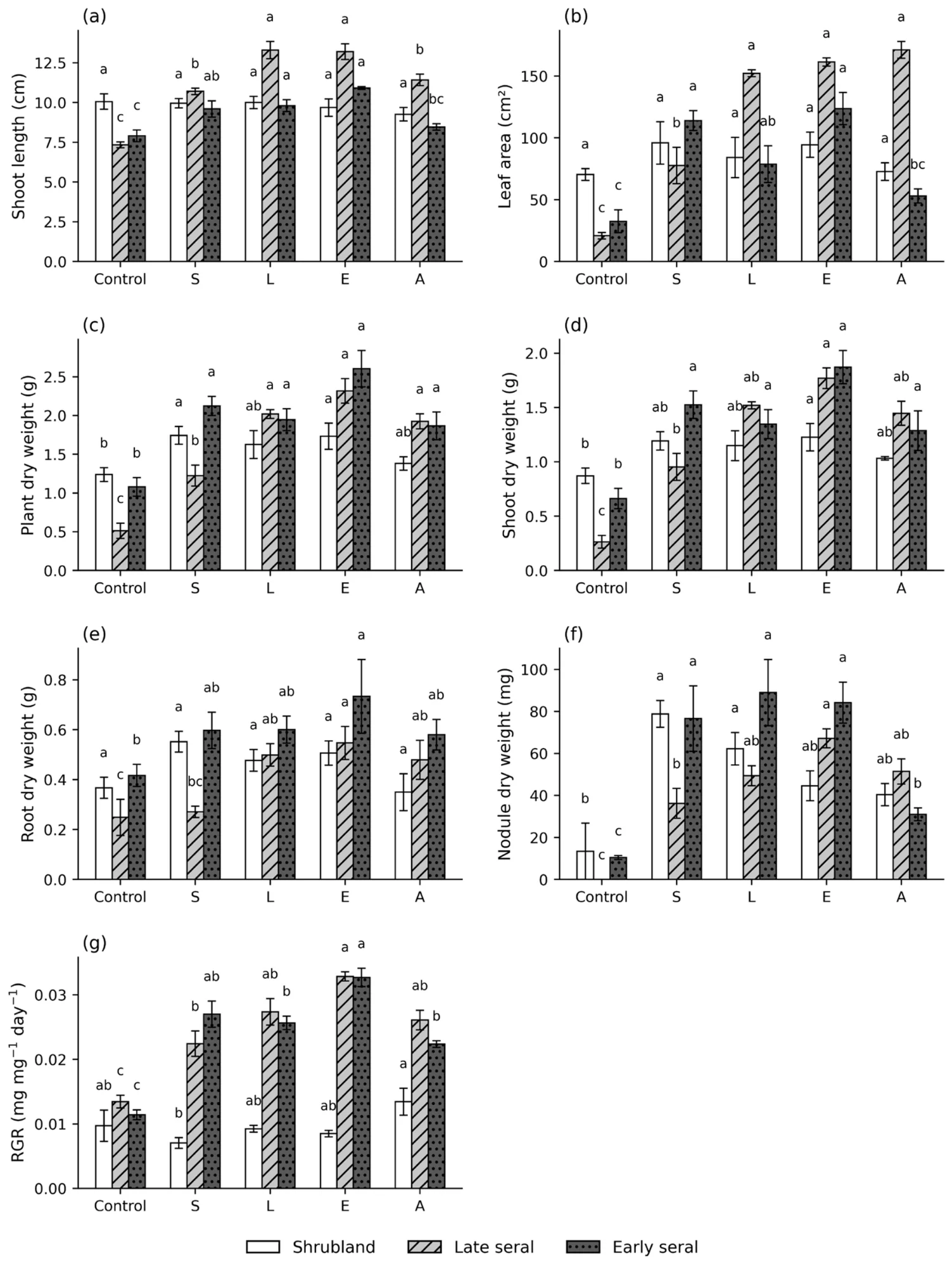
Effects of AMF inoculation on growth and growth-analysis traits of *Piscidia carthagenensis* grown in shrubland, early-seral, and late-seral soils. (**a**) Shoot length, (**b**) leaf area, (**c**) plant dry weight, (**d**) shoot dry weight, (**e**) root dry weight, (**f**) nodule dry weight, and (**g**) relative growth rate (RGR). Bars represent means ± SE (*n* = 5 per soil × inoculation combination). Different letters indicate significant differences among inoculation treatments within each recipient soil based on estimated marginal means with Tukey-adjusted comparisons following generalized linear models (GLMs) fitted with a Gamma error distribution and log link (*p* < 0.05). SE = standard error.

The effects of AMF inoculation on plant growth differed markedly among the three recipient soils (Figure 1; Table S1). Native AMF consortia generally produced stronger and more consistent positive responses than the tested non-native *Acaulospora lacunosa* inoculum, particularly in early- and late-seral soils.

In reference shrubland soil, plant responses to AMF inoculation were comparatively limited. Plant dry weight was significantly higher than the uninoculated control in plants inoculated with the native shrubland (S) and early-seral (E) AMF consortia (Figure 1c), whereas shoot dry weight increased significantly only with the early-seral consortium (E) (Figure 1d). No significant differences from the control were detected for root dry weight, shoot length, leaf area, or RGR. Nodule dry weight was significantly higher than the control in plants inoculated with the native shrubland (S) and late-seral (L) consortia.

Plants grown in late-seral soil showed pronounced responses to AMF inoculation. All inoculation treatments increased shoot length, leaf area, plant dry weight, shoot dry weight, nodule dry weight, and RGR relative to the uninoculated control. Root dry weight showed a more restricted response, increasing only in plants inoculated with the native late-seral (L) and early-seral (E) AMF consortia and with the *A. lacunosa* inoculum (A). Among inoculated treatments, the native late-seral (L) and early-seral (E) consortia generally produced the highest values across growth variables.

In early-seral soil, AMF inoculation promoted positive responses across several growth traits. Plant dry weight, shoot dry weight, nodule dry weight, and RGR increased under all inoculation treatments relative to the uninoculated control, whereas root dry weight increased significantly only in plants inoculated with the native early-seral consortium (E). Shoot length and leaf area increased only in plants receiving native AMF consortia, whereas the *A. lacunosa* inoculum did not significantly affect these traits. Among the native inocula, the early-seral consortium (E) produced significant positive responses across all growth variables evaluated.

### 2.3. Effects of Matching Inoculum Origin with Recipient Soil on Plant Performance

Home–Away comparisons revealed a trait-specific Home advantage (Table 3). Shoot dry weight, leaf area, and nodule dry weight were significantly greater under Home than Away inoculations, whereas plant dry weight, root dry weight, shoot length, and relative growth rate (RGR), did not differ significantly between these categories. Compared with the non-native *Acaulospora lacunosa* inoculum, Home inoculations resulted in significantly greater plant dry weight, shoot dry weight, root dry weight, leaf area, nodule dry weight, and shoot length. In contrast, RGR did not differ significantly between Home and non-native inoculations (Table 3).

### 2.4. Mycorrhizal Colonization Patterns Across Recipient Soils and Inoculum Sources

**Figure 2:**
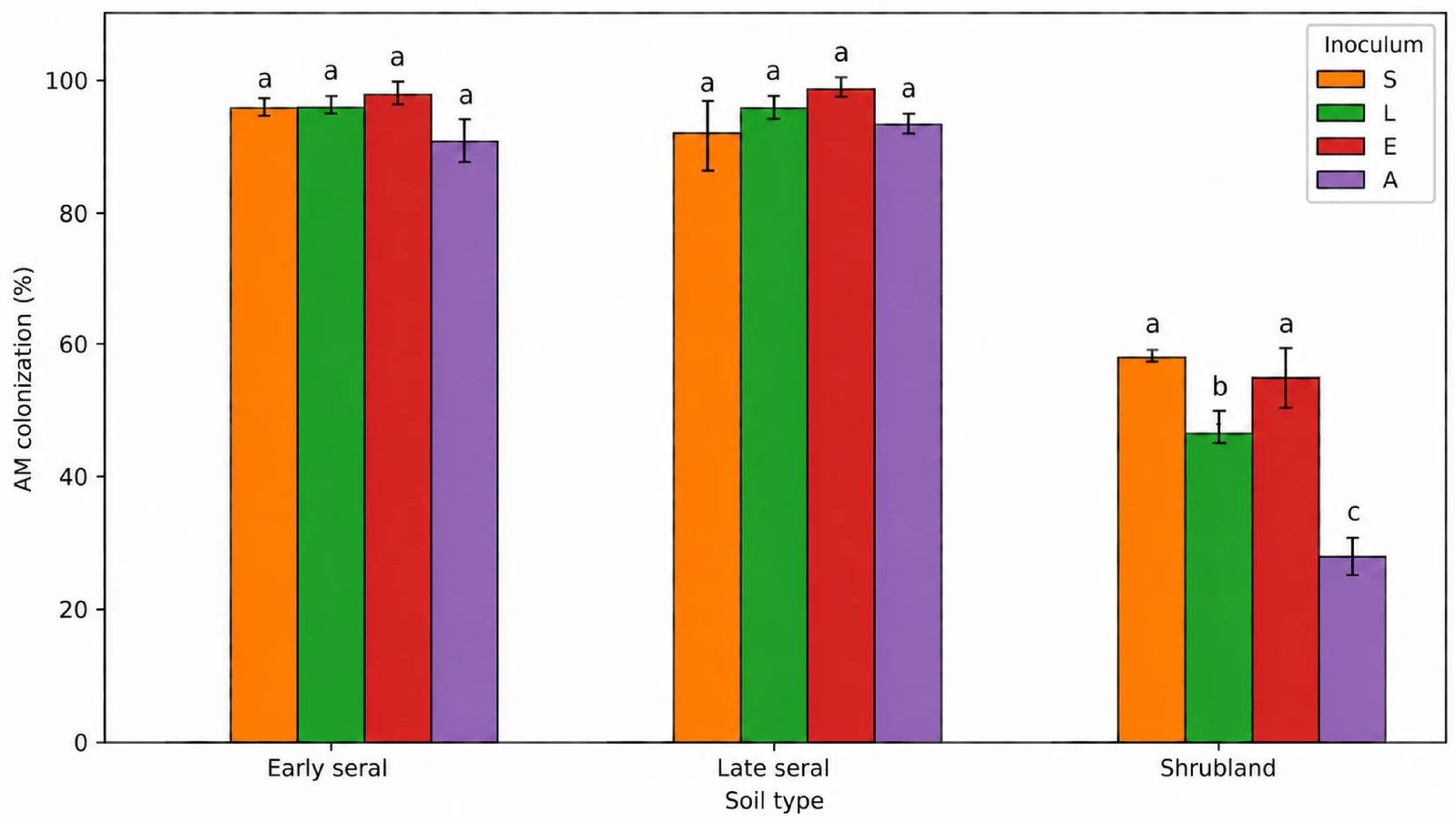
Arbuscular mycorrhizal (AM) root colonization of *Piscidia carthagenensis* under different inoculation treatments in reference shrubland, early-seral, and late-seral soils at 112 days. Values are means ± SE (*n* = 5). Different letters indicate significant differences among inoculated treatments within each soil (Tukey’s HSD, *p* < 0.05). Control plants were excluded from post hoc comparisons because colonization was zero. Control = uninoculated; S = native shrubland consortium; E = native early-seral consortium; L = native late-seral consortium; A = non-native *Acaulospora lacunosa* inoculum. SE = standard error. HSD = honestly significant difference.

AM colonization was successfully established in all inoculated treatments, whereas no colonization was detected in uninoculated controls in any recipient soil (Figure 2; Table 4), indicating the absence of detectable background AMF colonization. Two-way analysis of variance (ANOVA) revealed significant effects of recipient soil and inoculum source on total root colonization and all intraradical structures evaluated. The recipient soil × inoculum interaction was significant for arbuscules, vesicles, and hyphae, but not for total root colonization (Table S2).

Total root colonization differed markedly among recipient soils. In early- and late-seral soils, colonization exceeded 90% in all inoculated treatments and did not differ significantly among inoculum sources. In reference shrubland soil, colonization was lower and differed among inoculum sources: the native shrubland (S) and early-seral (E) consortia produced the highest colonization, followed by the late-seral consortium (L), whereas the non-native *Acaulospora lacunosa* inoculum (A) produced the lowest colonization (Figure 2; Table 4).

Intraradical structures showed more pronounced differences among inoculum sources than total colonization (Table 4). In early-seral soil, arbuscule abundance was highest with the native early-seral (E) and late-seral (L) consortia, intermediate with the shrubland consortium (S), and lowest with the *A. lacunosa* inoculum (A). In late-seral soil, all native consortia produced greater arbuscule abundance than A, with E showing the highest values and L an intermediate response among the native inocula. In reference shrubland soil, E and L produced greater arbuscule abundance than A, whereas S showed an intermediate response. Vesicle and hyphal abundance also differed among inoculum sources, with patterns varying among recipient soils (Table 4).

After accounting for recipient soil and inoculum effects, several weak-to-moderate correlations were observed between plant-performance and AMF-colonization variables; however, none remained significant after false discovery rate (FDR) correction (Table S4).

## 3. Discussion

### 3.1. Plant Responsiveness to AMF Differs Among Soils Representing Contrasting Successional Stages

Plant responses to AMF inoculation differed markedly among recipient soils representing contrasting stages of secondary succession. Native AMF consortia produced relatively modest growth responses in the reference shrubland soil, whereas their effects were more pronounced in early- and late-seral soils. These contrasting responses are consistent with the context dependency of plant–AMF interactions, whereby plant responsiveness to inoculation varies with both partner identity and environmental conditions [21]. Voříšková et al. [22] experimentally showed that abiotic conditions can strongly influence mycorrhizal functioning independently of changes in AMF community composition. In the same region of the Macanao Peninsula, Cáceres-Mago et al. [20] found species-specific responses of native woody plants to native AMF enrichment, with effects on growth and physiological traits varying among host species.

The recipient soils represented contrasting stages of secondary succession following sand extraction, a disturbance that removed the original vegetation and part of the upper soil horizon. These stages also differed in several physicochemical soil properties, providing distinct edaphic contexts for AMF–plant interactions. Differences in soil nutrient availability may provide one plausible explanation for the contrasting magnitude of plant responses among recipient soils. Plant nutritional status can strongly regulate AM symbiosis, with P and N availability acting as major determinants of mycorrhizal development and functioning [23]. In particular, the establishment, development, and maintenance of AM symbiosis are strongly influenced by plant P status [24]. Experimental evidence also indicates that P availability can influence plant growth responses to AMF. In *Medicago* spp., mycorrhizal growth response declined with increasing P supply [25]. Under low P availability (<10 mg kg^−1^ water-extractable P), AM symbiosis also improved P acquisition and stimulated biological N fixation, whereas benefits to P nutrition diminished or disappeared above this level. More recently, Pu et al. [26] showed that growth responsiveness to AMF varied across an Olsen-P gradient of 4, 18, and ≥60 mg kg^−1^ in maize, cotton, and wheat and was jointly shaped by soil P availability and host traits, generally peaking at intermediate P availability. A meta-analysis of 187 studies further showed that AMF inoculation typically increases plant biomass and N and P nutrition, while the magnitude of these responses varies with multiple biotic and abiotic factors, including nutrient conditions [27]. In our study, Bray-extractable P declined from 46.7 mg kg^−1^ in the reference shrubland soil to 27.8 and 7.6 mg kg^−1^ in the late- and early-seral soils, respectively, while total N declined from 0.40% to 0.16% and 0.06%. This nutrient gradient coincided with stronger growth responses to AMF in the seral soils, although its contribution to mycorrhizal responsiveness remains uncertain because nutrient availability was not experimentally manipulated.

### 3.2. Native AMF Consortia Generally Outperform the Tested Non-Native Isolate

Native AMF consortia generally produced stronger plant responses than the tested non-native *Acaulospora lacunosa* inoculum, particularly in the seral soils. Although *A. lacunosa* successfully established symbiosis and improved several plant traits, its effects were less consistent than those of the native consortia. Similar patterns have been reported in other woody species. Soufiani et al. [28] found that an indigenous multispecies AMF consortium promoted greater growth and mineral nutrition in *Argania spinosa* seedlings than a pure commercial strain of *Rhizophagus irregularis*, including under drought stress. Similarly, Godoy et al. [29] reported greater shoot and root biomass in *Araucaria araucana* seedlings inoculated with a native multispecies AMF inoculum than with single- or multispecies commercial inocula under greenhouse conditions. In legumes, Bencherif et al. [30] found that a native AMF inoculum from semiarid saline soils enhanced shoot biomass, mycorrhizal colonization, and shoot P content relative to a commercial inoculum across several *Medicago* and *Trifolium* species. Evidence across these studies supports the potential of native AMF inocula to promote plant performance across different hosts and environmental conditions, although their effects remain dependent on the particular inoculum–host–soil context.

Several non-exclusive factors may have contributed to the generally greater plant responses observed with native consortia than with the tested non-native isolate. Multispecies AMF assemblages can provide functional benefits not achieved by individual fungal isolates. Functional complementarity among AMF species has been demonstrated through enhanced host P acquisition by fungal mixtures relative to their component species [31]. Similarly, mixtures of native AMF from different families enhanced plant biomass and mineral nutrition more effectively than single-species inoculations under multiple edaphic stresses [32]. These functional outcomes may depend not only on species richness but also on community composition. AMF communities with contrasting life-history traits differed in their effects on host nutrient acquisition and plant growth [33], while the relative abundance of AMF species within multispecies assemblages influenced mycorrhizal functioning and plant growth [34]. In our study, all native inocula shared *Claroideoglomus* cf. *etunicatum* as the dominant morphotype but retained distinct assemblages of lower-abundance morphotypes, which may have contributed to their contrasting effects on plant performance. However, because inoculum composition was not experimentally manipulated and plant physiological responses were not measured, the mechanisms underlying treatment differences cannot be resolved. Moreover, native treatments were multispecies consortia whereas the non-native treatment was a single *Acaulospora lacunosa* isolate, preventing separation of inoculum origin from fungal richness and identity. Differences in inoculum mass also introduced potential variation in carrier-associated nutrients and microorganisms, despite standardization of total substrate mass. Therefore, the greater responses observed with native consortia should be interpreted as differences among the inocula tested rather than as an effect of native origin alone.

### 3.3. Intraradical Colonization Structures Provide Additional Information Beyond Total Colonization

Our results indicate that total root colonization alone did not reflect the differences in plant responses observed among AMF inoculation treatments. Although total colonization exceeded 90% across inoculation treatments in the two seral soils, plant responses differed among inocula. This pattern is consistent with evidence showing that the percentage of root length colonized does not necessarily predict plant growth or nutrient acquisition [35]. More recent experimental evidence further shows that relationships between AMF colonization and plant responses depend on the host, response trait, and colonization metric, and that greater total or arbuscular colonization does not necessarily translate into greater plant growth [36]. Differences among inocula in our study were more evident in intraradical structures, as arbuscule, vesicle, and hyphal abundance varied significantly and showed recipient soil × inoculum interactions. In particular, native consortia generally produced greater arbuscule abundance than the *Acaulospora lacunosa* inoculum in the seral soils.

Arbuscules form a major interface for reciprocal nutrient exchange between AMF and their hosts [37]. At this interface, fungal-acquired mineral nutrients are transferred to the plant in exchange for plant-derived carbon compounds [38], with transport systems associated with the periarbuscular membrane mediating P and N exchange [39]. However, the abundance of these structures should not be interpreted as a direct measure of symbiotic function. Arbuscules are ephemeral, and their abundance at a single sampling point may not reflect cumulative exchange activity [36]. Accordingly, greater arbuscule abundance with some native consortia indicates differences in intraradical development, but not necessarily greater nutrient transfer.

AMF inoculation was also associated with increased nodule biomass in several treatments, with native consortia producing the most consistent positive responses across recipient soils. This pattern is consistent with broader evidence of interactions between mycorrhizal and rhizobial symbioses in legumes. A global meta-analysis showed that AMF and/or rhizobial inoculation increased nodule number and weight, together with plant N and P content and shoot biomass, although responses varied with environmental conditions [40]. Recent evidence further indicates that AMF can positively regulate nodulation in legume–rhizobium–AMF associations, although outcomes depend on symbiont identity and environmental conditions [41]. In the present study, the microbial wash was used to reduce differences in the non-AMF microbial background among treatments, but it could not standardize microorganisms introduced with each inoculum. Therefore, residual differences in inoculum-associated microorganisms, including rhizobia, may have contributed to variation in nodulation, and the observed changes in nodule biomass cannot be attributed solely to AMF.

### 3.4. Limited Effects of Matching Inoculum Origin with Recipient Soil on Plant Performance

Home–Away comparisons revealed limited and trait-specific advantages of matching native AMF inocula with recipient soils corresponding to their successional stage of origin. Home inoculations resulted in greater shoot dry weight, leaf area, and nodule dry weight than Away inoculations, whereas plant dry weight, root dry weight, shoot length, and RGR, did not differ between these categories. Plant responses to AMF can vary with the geographic origin of the interacting partners and soil [16], while resource availability and local environmental conditions may further influence the expression of these relationships [42,43]. In our study, the Home advantage was restricted to specific traits, indicating that matching inoculum origin with recipient soil can benefit some plant responses, but providing no support for a generalized Home advantage.

A clearer pattern emerged when Home inoculations were compared with the tested non-native *Acaulospora lacunosa* inoculum. Home inoculations produced greater plant, shoot, and root dry weight, leaf area, nodule dry weight, and shoot length, whereas RGR did not differ. Thus, Home inoculations outperformed the non-native isolate for six of the seven traits evaluated, compared with only three of seven in the Home–Away comparison. This broader advantage of native inocula may be particularly relevant for restoration, as native AMF have been shown to promote plant establishment and successional recovery in degraded ecosystems [44]. Our results therefore suggest that native AMF consortia may be promising candidate inocula for restoration, without indicating that their successional origin must necessarily match that of the recipient soil.

Finally, a limitation of this study is that, although soils and native AMF inocula were collected independently from three sites within each successional stage, they were subsequently pooled within stage for the greenhouse experiment. Therefore, successional origin was not independently replicated, and the observed responses should be interpreted as applying to the specific composite soils and inocula evaluated rather than as general effects of successional stage.

## 4. Materials and Methods

### 4.1. Study Area

**Figure 3:**
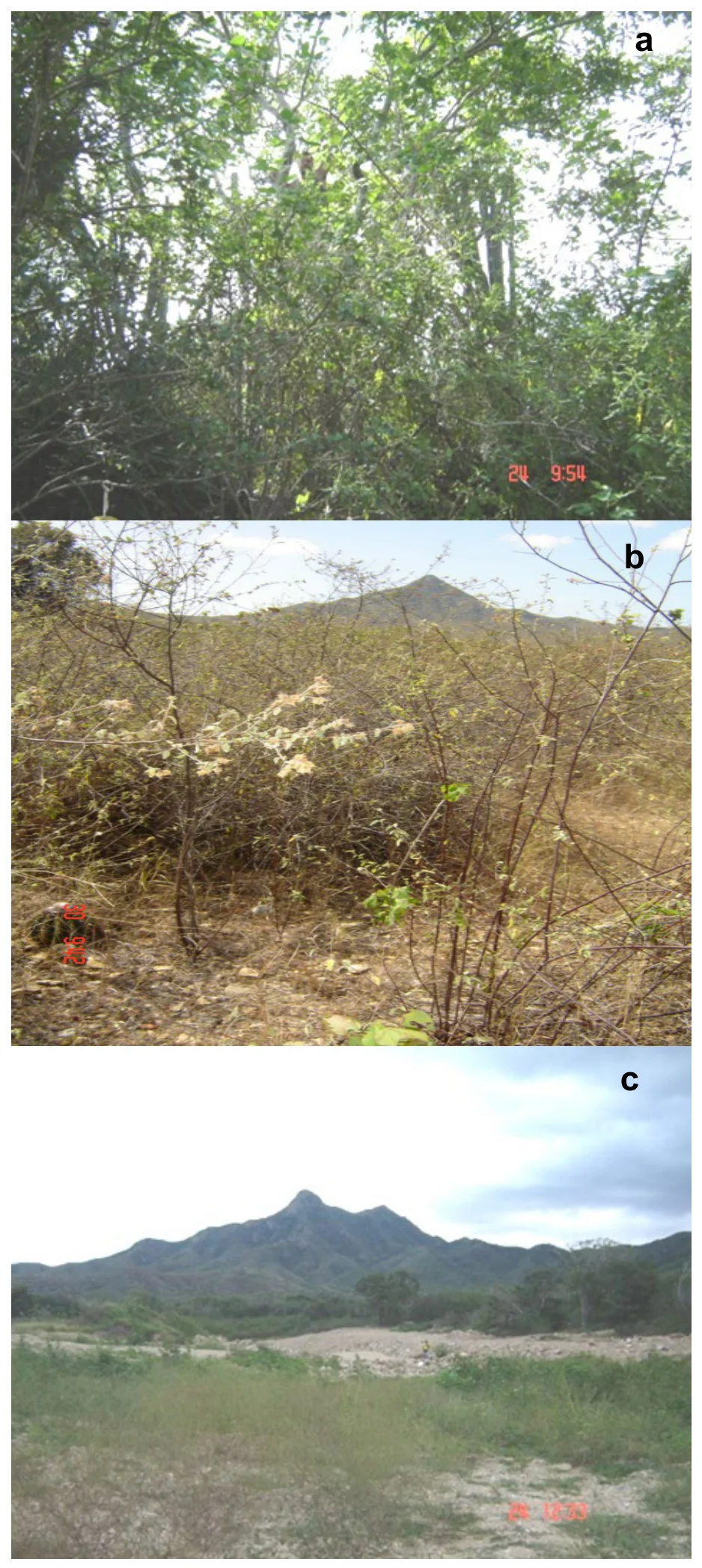
Successional mosaic of xerophytic shrubland in Macanao, Margarita Island, Venezuela. (**a**) Reference xerophytic shrubland; (**b**) late-seral site (20 years after sand extraction); (**c**) early-seral site (2 years after sand extraction).

The successional mosaic resulting from sand mining in the vicinity of the Quebrada La Chica (11°01′19″–11°04′09″ N; 64°16′36″–64°15′08″ W), located in the Peninsula de Macanao, Margarita Island, Venezuela, was the source of the soils and native AMF used in the present study. Annual rainfall ranges from 326 to 522 mm with a seasonal distribution throughout the year: rainfall peaks in August, and a second, smaller rainfall peak occurs at the end of the year. The dry season runs from January to June, and the annual average temperature is 27.4 °C [45]. The selected successional stages included an early-seral plant community, established two years after mining cessation, and a late-seral plant community, established 20 years after mining cessation (Figure 3). A xerophytic shrubland was selected as the reference ecosystem. This reference vegetation is characterized by native woody species representative of the drylands of the Macanao Peninsula, including *Croton flavens*, *Capparis odoratissima*, *Cercidium praecox*, and *Stenocereus griseus* [17].

### 4.2. Plant Material

*Piscidia carthagenensis* Jacq. is a leguminous tree distributed from Mexico through Central America and the Caribbean to northern and western South America, including Venezuela, Colombia, Ecuador, and Peru [46]. In the Macanao Peninsula, the species occurs naturally in disturbed riparian habitats affected by sand extraction and is associated with deciduous forests, thorn shrublands, and sites at different stages of secondary succession [17]. Mature seeds of *Piscidia carthagenensis* were collected between March and April from adult trees growing along disturbed intermittent streambeds in the Macanao Peninsula. This collection period coincides with the species’ fruiting season, which follows flowering during the dry season.

### 4.3. Soil Collection and Analysis

Surface soils (0–20 cm depth) were collected from three independent locations within each successional stage: reference shrubland, late-seral, and early-seral communities. At each location, approximately six to seven soil cores were collected and homogenized to obtain one composite sample per location. Thus, three independent composite samples were obtained for each successional stage and were used for physicochemical characterization (*n* = 3 per stage). For the greenhouse bioassay, the three composite soil samples corresponding to each successional stage were subsequently pooled and homogenized to generate one recipient soil per stage. Consequently, successional stage was not treated as an independently replicated experimental factor in the greenhouse analysis; instead, inoculation effects were evaluated within each pooled recipient soil.

Soil physicochemical characterization included organic matter determined by the Walkley and Black method [47]; total nitrogen determined by the micro-Kjeldahl method [48], and total and available phosphorus determined following Bray and Kurtz [49]. Available P was extracted with NH_4_F and HCl, and phosphorus in both determinations was quantified colorimetrically using the molybdate–ascorbic acid method [50]. Soil texture was determined according to [51], and pH was measured in water using the potentiometric method [47].

A portion of each of the three site-level composite soil samples collected within each successional stage was used to establish trap cultures with the highly mycorrhizal host plant *Vigna luteola*. Trap cultures were maintained for six months. After this period, irrigation was suspended for two weeks to further promote AMF sporulation [52]. Native inocula were initially prepared for each site using trap-culture substrate containing AMF-infected roots, spores, and hyphae. The inocula from the three sites within each successional stage were subsequently pooled and homogenized to produce one native AMF consortium per successional stage. Native AMF consortia were designated using a single-letter code corresponding to their successional stage of origin: reference shrubland (S), early-seral (E), and late-seral (L). The non-native inoculum used in this study, *Acaulospora lacunosa* (A), was produced by the Soil Ecology Laboratory of the Instituto Venezolano de Investigaciones Científicas (IVIC) for scientific and commercial purposes. This inoculum contained 6136 spores per 100 g of dry soil. *A. lacunosa* spores were not detected in any of the native inocula produced.

The native inoculants were taxonomically characterized based on spore morphology. Spores were isolated by wet sieving and centrifugation in a sucrose gradient, following Sieverding [53]. For each AMF spore morphotype, permanent slides were prepared using polyvinyl alcohol and lactoglycerol (PVLG) as mounting media. Morphological assignment of AMF spores was performed at the genus level and, when possible, at the species level, according to [54]. Taxonomic assignment was based on spore morphology and current taxonomic concepts available through the International Collection of (Vesicular) Arbuscular Mycorrhizal Fungi (INVAM, University of Kansas). No molecular identification or phylogenetic analysis was performed; therefore, the taxonomic identities reported here should be interpreted as morphology-based assignments.

### 4.4. Greenhouse Bioassay

The experiment followed a completely randomized design comprising three pooled recipient soils representing contrasting successional stages (reference shrubland, early-seral, and late-seral) and five inoculation treatments. As described above, the three site-level soil composites within each successional stage were pooled and homogenized before establishment of the greenhouse experiment. The inoculation treatments consisted of an uninoculated control (Control), three native AMF consortia originating from reference shrubland (S), early-seral (E), and late-seral (L) soils, and the non-native *Acaulospora lacunosa* inoculum (A). Inocula were designated by a letter code corresponding to their origin or identity, whereas recipient soils were identified by their full successional category.

Seedlings of *P. carthagenensis*, previously germinated in sterile sand, were transplanted into containers filled with 800 g of soil. Recipient soils were sterilized by gamma irradiation at a dose of 8 kGy. The amount of each AMF inoculum applied per plant was adjusted according to inoculum-specific spore density to provide approximately comparable spore numbers among inoculation treatments. The non-native *Acaulospora lacunosa* inoculum (A), containing 6136 spores per 100 g of dry substrate, was used as the reference; 25 g of inoculum was applied per plant, corresponding to approximately 1534 spores. The native inocula contained 961 ± 116, 1307.5 ± 110, and 1959.8 ± 363 spores per 100 g of dry substrate for the reference shrubland (S), late-seral (L), and early-seral (E) consortia, respectively. Based on these spore densities, approximately 160 g of S, 117 g of L, and 78 g of E inoculum were applied per plant, corresponding to estimated inputs of 1538, 1530, and 1529 spores, respectively. In all treatments, differences in inoculum mass were compensated for with the corresponding sterilized recipient soil so that all pots contained the same total substrate mass. Thus, inoculum mass was adjusted to provide a target of approximately 1530 AMF spores per plant across inoculation treatments. To reduce potential differences in the non-AMF microbial community among inoculation treatments within each recipient soil, each plant received 50 mL of a microbial wash prepared from fresh, non-sterilized soil corresponding to its respective recipient soil, following the approach of Sikes et al. [55]. The microbial wash was prepared by suspending soil in sterile water (1 kg L^−1^) and sequentially filtering the suspension through 38- and 20-µm sieves to exclude AMF propagules while retaining smaller soil microorganisms. This approach is commonly used in AMF experiments to reduce differences in the non-AMF microbial background, although complete standardization of microbial communities cannot be assumed [56]. Greenhouse conditions were maintained at approximately 23 °C and 90% relative humidity, with a 16 h light photoperiod. Plants were watered twice weekly with tap water to approximately field capacity, and no fertilizer was applied during the experiment.

Five plants per soil × inoculation combination were destructively harvested 30 days after planting to obtain the initial measurements required for growth analysis, and a second set of five plants per combination was harvested at 112 days for final plant-performance measurements. At both harvests, plants were separated into leaves, stems, and roots, and dry weight (DW) was determined after oven-drying at 60 °C to constant weight. Leaf area per plant was determined gravimetrically using millimeter-paper replicas of all leaves. At the final harvest, nodule dry weight and shoot height were also determined. Relative growth rate (RGR) was calculated over the 30–112 day interval following Hunt et al. [57] as RGR = (ln DW_2_ − ln DW_1_)/(t_2_ − t_1_), where DW_1_ and DW_2_ represent total plant dry weight at the initial and final destructive harvests, respectively, and t_1_ and t_2_ correspond to 30 and 112 days after planting. Plants from the two independent destructive harvests were paired using random numbers within each recipient soil × inoculation combination to calculate replicate-level RGR.

### 4.5. Quantification of AMF Colonization

Root colonization variables were assessed at the final harvest, 112 days after planting. Fine roots (≤2 mm in diameter) were cleared in 10% KOH for 30 min at 90 °C, rinsed three times with water, acidified in 10% HCl for 15 min at room temperature, and then stained with trypan blue for 15 min at 90 °C [58]. For each plant, 30 cm of stained roots were mounted on microscope slides using lactoglycerol (lactic acid, glycerol and water, 1:1:2). Slides were examined under a light microscope at 200× magnification to assess the presence of arbuscular mycorrhizal fungal (AMF) structures, including arbuscules, hyphal coils, vesicles, and aseptate hyphae. Colonization was quantified using the magnified intersect method [59], with AMF structures recorded at 100 intersections per sample. The occurrence of each intraradical structure was recorded independently; when more than one structure of the same category occurred at an intersection, it was counted once. Total AMF colonization was calculated as the percentage of intersections containing any AMF structure. Accordingly, the percentages of arbuscules, vesicles, and hyphae were independently quantified and were not treated as additive components of total colonization.

### 4.6. Statistical Analysis

All statistical analyses were performed in R (version 4.6.1; R Core Team, [60]). Generalized linear models (GLMs) were fitted using the stats package (version 4.6.1), and post hoc pairwise comparisons were performed on estimated marginal means using Tukey-adjusted comparisons. Data visualization was conducted using ggplot2 (version 4.0.3) [61]. Plant performance variables included total dry biomass, shoot biomass, root biomass, leaf area, shoot length, relative growth rate (RGR), and nodule dry weight.

To evaluate the effects of mycorrhizal inoculation on plant performance, separate GLMs were fitted for shrubland, early-seral, and late-seral recipient soils. Separate models were used because a single pooled recipient soil was used for each successional stage in the greenhouse experiment, thereby avoiding pseudoreplication that would arise from treating successional stage as an independently replicated experimental factor. Inoculation treatment was included as a fixed factor. Models assumed a Gamma error distribution with a logarithmic link function. Because this distribution requires strictly positive response values, a small positive constant corresponding to one-half of the minimum positive value within the respective dataset was added to nodule dry weight before model fitting when zero values were present. Model adequacy was evaluated using deviance residuals and dispersion statistics. Sensitivity to the choice of constant was assessed by refitting the nodule dry-weight models using constants corresponding to one-quarter and one-tenth of the minimum positive value. Overall inoculation effects were evaluated using Wald χ^2^ tests, and pairwise comparisons among inoculation treatments were conducted on estimated marginal means using Tukey-adjusted comparisons.

To evaluate whether matching between native AMF inoculum origin and recipient soil was associated with enhanced plant performance, native inoculation treatments were classified as Home when inoculum and recipient soil corresponded to the same successional stage and as Away when they originated from different successional stages. The non-native *Acaulospora lacunosa* inoculum was included as a third category (non-native). Plant performance variables were analyzed as a function of Match Category and recipient soil, both included as fixed factors, using the same Gamma GLM framework described above. Planned contrasts between Home and Away and between Home and non-native treatments were evaluated from estimated marginal means.

Root colonization variables (total colonization, arbuscules, vesicles, and hyphae) were analyzed using two-way analysis of variance (ANOVA), with recipient soil, inoculum source, and their interaction included as fixed factors. Percentage data were arcsine square-root-transformed prior to analysis. Uninoculated control plants were excluded from post hoc comparisons because AMF colonization was consistently zero. When significant main effects or interactions were detected, pairwise comparisons among inoculum treatments within each recipient soil were performed using Tukey’s honestly significant difference (HSD) test. Because one pooled recipient soil represented each successional stage in the greenhouse experiment, effects involving recipient soil were interpreted as differences among the specific pooled soils evaluated rather than as replicated effects of successional stage. Differences in soil physicochemical properties among successional stages were evaluated separately using one-way ANOVA based on the three independent site-level composite samples per successional stage. When significant differences were detected, means were compared using Tukey’s HSD test (*p* < 0.05).

Exploratory relationships among plant-performance, nodulation, and AMF-colonization variables were evaluated in inoculated plants using Spearman correlations on residuals obtained after accounting for recipient soil and inoculum effects; *p* values were adjusted for multiple comparisons using the false discovery rate (FDR).

A supplementary assessment of uncertainty in mean RGR combined the sampling variances of log-transformed initial and final biomass from the independent harvests. Conditional 95% confidence intervals were calculated using the Welch–Satterthwaite approximation (Table S3).

## 5. Conclusions

This study showed that the response of *Piscidia carthagenensis* to AMF inoculation varied among the recipient soils evaluated. Native AMF consortia generally promoted greater plant growth and nodulation than the tested non-native *Acaulospora lacunosa* inoculum. These effects were more evident in early- and late-seral soils. Matching inoculum origin with recipient soil benefited only specific plant traits and was not consistently advantageous across all performance variables. This suggests that strict correspondence between inoculum origin and recipient-soil successional stage may not be necessary to obtain positive responses. Differences among inocula were also more clearly expressed in intraradical structures than in total root colonization, supporting the complementary value of characterizing arbuscules, vesicles, and hyphae when evaluating AMF colonization. Because the native treatments consisted of multispecies AMF consortia, whereas the non-native treatment consisted of a single fungal isolate, their greater performance should be interpreted as a difference among the inocula tested rather than as an effect of native origin alone. Overall, the native AMF consortia evaluated here successfully established symbiosis and enhanced plant performance across the recipient soils tested, suggesting their potential as candidate inocula for restoration of degraded dryland ecosystems. However, because each successional stage was represented by one pooled recipient soil and one pooled native AMF consortium, and the experiment involved a single host species under greenhouse conditions, these findings require validation under independently replicated field conditions before broader application.

## Figures and Tables

**Table 1:** Physicochemical properties of soils representing three successional stages of tropical xerophytic shrubland affected by sand mining. Values are means ± SE (*n* = 3 independent sites per successional stage). Different letters within a column indicate significant differences among successional stages based on Tukey’s HSD test (*p* < 0.05). SE = standard error. HSD = honestly significant difference.

Seral Stages	Total P (mg kg^−1^)	Available P (mg kg^−1^)	Total N (%)	OM (%)	pH (H_2_O)	Soil Texture
Shrubland	446.7 (5.4) ^a^	46.7 (7.2) ^a^	0.40 (0.04) ^a^	1.7 (0.1) ^a^	6.6 (0.5) ^b^	Sandy loam
Late-seral	300.1 (20.3) ^b^	27.8 (4.8) ^b^	0.16 (0.01) ^b^	1.6 (0.3) ^a^	6.9 (0.1) ^b^	Loamy sand
Early-seral	169.9 (2.8) ^c^	7.6 (1.4) ^c^	0.06 (0.00) ^c^	1.1 (0.08) ^b^	7.5 (0.04) ^a^	Loamy sand

OM: organic matter; P: phosphorus; N: nitrogen. Soil texture was classified according to the USDA soil texture classification system.

**Table 2:** Relative contribution of arbuscular mycorrhizal fungal (AMF) morphotypes in three native trap-culture inocula: shrubland (S), late-seral (L), and early-seral (E). Contribution levels are study-specific descriptive classes based on the total number of spores recorded across three 50 g subsamples per inoculum: >50 (high), 20–50 (medium), 5–20 (low), and <5 (rare). A dash (–) indicates that the morphotype was not detected. Taxonomic names represent morphology-based assignments from the original spore characterization.

No.	Morphology-Based Taxonomic Assignment	S	L	E
1	*Claroideoglomus* cf. *etunicatum* (Claroideoglomeraceae)	medium	high	high
2	*Entrophospora infrequens* (Entrophosporaceae)	low	medium	high
3	*Funneliformis geosporum* (Glomeraceae)	–	high	medium
4	*Diversispora spurca* (Diversisporaceae)	low	–	rare
5	*Acaulospora* cf. *laevis* (Acaulosporaceae)	–	medium	–
6	*Funneliformis mosseae* (Glomeraceae)	–	low	–
7	*Glomus* cf. *tortuosum* (Glomeraceae)	low	medium	–
8	*Glomus aggregatum* (Glomeraceae)	medium	low	–
9	*Sclerocystis sinuosa* (Glomeraceae)	medium	–	–
10	*Sclerocystis* sp. 1 (Glomeraceae)	rare	–	–
11	*Sclerocystis* sp. 2 (Glomeraceae)	low	–	–
12	*Glomus* s.l. (yellow thin wall)	low	–	low
13	*Glomus* s.l. (yellow coarse wall)	–	–	rare
14	*Glomus* s.l. *Glomus* sp. 1	medium	high	–
15	*Glomus* s.l. *Glomus* sp. 2	medium	low	–
16	*Glomus* s.l. (thin red filament)	rare	–	–
17	*Glomus* s.l. (yellow filament)	low	–	–
18	*Glomus* s.l. (yellow multiple walls)	rare	–	–
19	Yellow with bulb (Gigasporaceae)	high	–	–
20	Yellow-orange with bulb (Gigasporaceae)	medium	–	–

Note: S, L, and E are native AMF consortia; A corresponds to the non-native monospecific *Acaulospora lacunosa* inoculum and is not included.

**Table 3:** Home–Away and Home–non-native contrasts for growth and growth-analysis traits of *Piscidia carthagenensis*. Estimates were obtained from generalized linear models (GLMs) fitted with a Gamma error distribution and log link and are presented as log-scale contrast estimates (± SE). Positive estimates indicate greater trait values under Home inoculation than under the corresponding comparison category. Asterisks denote significance levels: *p* < 0.05 (*), *p* < 0.01 (**), and *p* < 0.001 (***); ns, not significant. SE = standard error.

Trait	Home vs. Away	Home vs. Non-Native
Plant dry weight	0.121 (0.067) ns	0.256 (0.065) ***
Shoot dry weight	0.143 (0.067) *	0.309 (0.067) ***
Root dry weight	0.097 (0.068) ns	0.179 (0.067) **
Leaf area	0.136 (0.067) *	0.301 (0.066) ***
Nodule dry weight	0.214 (0.067) **	0.286 (0.067) ***
Shoot length	0.123 (0.067) ns	0.238 (0.067) ***
Relative Growth Rate (RGR)	−0.008 (0.021) ns	−0.036 (0.019) ns

**Table 4:** AM root colonization and intraradical structures of *Piscidia carthagenensis* grown in reference shrubland, early-seral, and late-seral recipient soils under different AMF inoculation treatments. Values are means ± SE (*n* = 5 per soil × inoculation combination). Different letters indicate significant differences among inoculation treatments within each recipient soil according to Tukey’s HSD test (*p* < 0.05). Uninoculated controls were excluded from post hoc comparisons because AM colonization was consistently zero. Control = uninoculated; S = native shrubland AMF consortium; E = native early-seral AMF consortium; L = native late-seral AMF consortium; A = non-native *Acaulospora lacunosa* inoculum. SE = standard error. HSD = honestly significant difference.

Recipient Soil	Inoculant	AM Colonization (%)	Arbuscules (%)	Vesicles (%)	Hyphae (%)
Shrubland	Control	0.0 (0.0)	0.0 (0.0)	0.0 (0.0)	0.0 (0.0)
	S	58.0 (0.3) ^a^	17.6 (0.5) ^ab^	12.8 (0.3) ^b^	27.6 (0.4) ^a^
	L	46.6 (1.3) ^b^	20.0 (0.7) ^a^	10.4 (0.5) ^b^	16.2 (0.7) ^b^
	E	55.1 (4.5) ^a^	22.2 (0.7) ^a^	14.2 (1.1) ^a^	18.7 (2.8) ^b^
	A	28.0 (2.7) ^c^	15.2 (0.6) ^b^	6.4 (0.6) ^c^	6.4 (1.1) ^c^
Late seral	Control	0.0 (0.0)	0.0 (0.0)	0.0 (0.0)	0.0 (0.0)
	S	92.0 (5.5) ^a^	61.6 (0.9) ^b^	12.4 (1.2) ^b^	18.0 (2.1) ^b^
	L	95.8 (1.5) ^a^	65.0 (1.3) ^ab^	15.6 (1.2) ^ab^	15.2 (1.1) ^b^
	E	98.5 (0.4) ^a^	67.6 (0.9) ^a^	17.6 (0.7) ^a^	13.3 (0.7) ^b^
	A	93.4 (1.4) ^a^	40.7 (2.0) ^c^	18.0 (1.0) ^a^	34.7 (2.1) ^a^
Early seral	Control	0.0 (0.0)	0.0 (0.0)	0.0 (0.0)	0.0 (0.0)
	S	95.9 (1.2) ^a^	60.0 (2.0) ^b^	17.2 (1.1) ^a^	18.7 (0.8) ^b^
	L	96.0 (1.0) ^a^	67.6 (0.7) ^a^	17.6 (0.7) ^a^	10.9 (0.6) ^c^
	E	97.8 (1.3) ^a^	72.2 (1.2) ^a^	16.0 (0.9) ^b^	9.6 (0.6) ^c^
	A	90.9 (3.2) ^a^	45.6 (2.4) ^c^	18.4 (1.1) ^a^	26.9 (2.4) ^a^

## Data Availability

The data supporting the findings of this study are available from the corresponding author upon reasonable request.

## Supplementary Materials

The following supporting information can be downloaded at: https://www.mdpi.com/article/10.3390/plants15213386/s1, Table S1: Overall effects of arbuscular mycorrhizal fungal (AMF) inoculation on growth and growth-analysis traits of *Piscidia carthagenensis* grown in shrubland, late-seral, and early-seral recipient soils; Table S2: Two-way analysis of variance (ANOVA) evaluating the effects of recipient soil, AMF inoculum source, and their interaction on total root colonization and intraradical structures of *Piscidia carthagenensis*; Table S3: Mean relative growth rate (RGR), standard errors (SEs), and 95% confidence intervals (CIs) for the 15 recipient soil × inoculation combinations over the 30–112-day interval. Five plants were harvested per combination at each independent destructive harvest. Standard errors combine the sampling variances of log-transformed initial and final biomass. Confidence intervals were calculated using the Welch–Satterthwaite approximation; Table S4: Exploratory Spearman correlations among plant-performance, nodulation, and AMF-colonization variables in inoculated plants (*n* = 60), based on residuals after accounting for recipient soil and inoculum effects. *p* values were adjusted using the false discovery rate (FDR).

## Abbreviations

The following abbreviations are used in this manuscript:

AMF	arbuscular mycorrhizal fungi
RGR	relative growth rate
DW	dry weight
ANOVA	analysis of variance
GLM	generalized linear model
HSD	honestly significant difference
CI	confidence interval
FDR	false discovery rate
