# Supplementary material for: Responses of Piscidia carthagenensis to Native and Non-Native AMF Inoculation Across Successional Soils from a Xerophytic Shrubland
Source: Plants (Basel). 2026 Nov 1;15(21):3386. doi: 10.3390/plants15213386 (PMC13766084; doi:10.3390/plants15213386)
Supplement: Supplementary file 1 [file plants-15-03386-s001.zip › plants-4591339-supplementary.pdf]

Table S1. Overall effects of arbuscular mycorrhizal fungal (AMF) inoculation on growth and growth-analysis traits of *Piscidia carthagenensis* grown in shrubland, late-seral, and early-seral recipient soils. Values correspond to Wald χ^2 tests from generalized linear models (GLMs) fitted separately for each recipient soil using a Gamma error distribution with a log link. Inoculation treatment was included as a fixed factor. Significant effects indicate differences among inoculation treatments

Response variable	Shrubland	Late-seral	Early-seral
Plant dry weight	$\chi^2(4)=16.02$, $P=0.003$	$\chi^2(4)=150.94$, $P<0.001$	$\chi^2(4)=62.58$, $P<0.001$
Shoot dry weight	$\chi^2(4)=10.64$, $P=0.031$	$\chi^2(4)=159.95$, $P<0.001$	$\chi^2(4)=48.33$, $P<0.001$
Root dry weight	$\chi^2(4)=10.07$, $P=0.039$	$\chi^2(4)=19.56$, $P<0.001$	$\chi^2(4)=9.68$, $P=0.046$
Nodule dry weight	$\chi^2(4)=16.02$, $P=0.003$	$\chi^2(4)=363.25$, $P<0.001$	$\chi^2(4)=165.38$, $P<0.001$
Leaf area	$\chi^2(4)=4.33$, $P=0.364$	$\chi^2(4)=293.09$, $P<0.001$	$\chi^2(4)=42.88$, $P<0.001$
Shoot length	$\chi^2(4)=2.35$, $P=0.672$	$\chi^2(4)=233.06$, $P<0.001$	$\chi^2(4)=45.93$, $P<0.001$
Relative growth rate (RGR)	$\chi^2(4)=10.48$, $P=0.033$	$\chi^2(4)=101.92$, $P<0.001$	$\chi^2(4)=227.94$, $P<0.001$

Table S2. Two-way analysis of variance (ANOVA) evaluating the effects of recipient soil, AMF inoculum source, and their interaction on total root colonization and intraradical structures of *Piscidia carthagenensis*. Analyses were performed on arcsine square-root-transformed percentage data. Values represent *F* statistics and associated *P* values.

Variable	Effect	*F*	*P*
Total colonization	Soil	288.59	<0.001
	Inoculum	12.08	<0.001
	Soil × Inoculum	2.19	0.061
Arbuscules	Soil	1376.71	<0.001
	Inoculum	137.54	<0.001
	Soil × Inoculum	14.04	<0.001
Vesicles	Soil	113.73	<0.001
	Inoculum	60.36	<0.001
	Soil × Inoculum	23.72	<0.001
Hyphae	Soil	130.82	<0.001
	Inoculum	27.38	<0.001
	Soil × Inoculum	3.95	0.0028

Table S3. Mean relative growth rate (RGR), standard errors (SEs), and 95% confidence intervals (CIs) for the 15 recipient soil × inoculation combinations over the 30–112-day interval. Five plants were harvested per combination at each independent destructive harvest. Standard errors combine the sampling variances of log-transformed initial and final biomass. Confidence intervals were calculated using the Welch–Satterthwaite approximation.

Recipient soil	Inoculum	Mean RGR (day^{-1})	SE (day^{-1})	95% CI (day^{-1})
Shrubland	Control	0,009728	0,003247	0.001307–0.018149
Shrubland	S	0,007037	0,001329	0.003933–0.010142
Shrubland	L	0,009245	0,001877	0.004890–0.013600
Shrubland	E	0,008491	0,001871	0.004176–0.012807
Shrubland	A	0,013436	0,002582	0.006676–0.020196
Late seral	Control	0,013446	0,003701	0.004906–0.021986
Late seral	S	0,022418	0,003008	0.014994–0.029841
Late seral	L	0,027362	0,002132	0.021602–0.033122
Late seral	E	0,032848	0,001617	0.029064–0.036632
Late seral	A	0,026094	0,001674	0.021605–0.030582
Early seral	Control	0,011412	0,001496	0.007885–0.014938
Early seral	S	0,026995	0,002367	0.020813–0.033178
Early seral	L	0,025655	0,001103	0.023026–0.028284
Early seral	E	0,032686	0,001255	0.029522–0.035851
Early seral	A	0,022371	0,001356	0.019241–0.025500

Table S4. Exploratory Spearman correlations among plant-performance, nodulation, and AMF-colonization variables in inoculated plants (n = 60), based on residuals after accounting for recipient soil and inoculum effects. P values were adjusted using the false discovery rate (FDR).

Plant variable	Total colonization	Arbuscules	Vesicles	Hyphae
Shoot length	ϱ = 0.107; *P*_FDR = 0.899	ϱ = −0.017; *P*_FDR = 0.984	ϱ = 0.055; *P*_FDR = 0.899	ϱ = −0.009; *P*_FDR = 0.984
Leaf area	ϱ = 0.168; *P*_FDR = 0.700	ϱ = −0.108; *P*_FDR = 0.899	ϱ = 0.294; *P*_FDR = 0.160	ϱ = 0.003; *P*_FDR = 0.984
Plant dry weight	ϱ = 0.363; *P*_FDR = 0.088	ϱ = −0.076; *P*_FDR = 0.899	ϱ = −0.064; *P*_FDR = 0.899	ϱ = 0.074; *P*_FDR = 0.899
Shoot dry weight	ϱ = 0.256; *P*_FDR = 0.268	ϱ = −0.067; *P*_FDR = 0.899	ϱ = −0.041; *P*_FDR = 0.919	ϱ = 0.061; *P*_FDR = 0.899
Root dry weight	ϱ = 0.349; *P*_FDR = 0.088	ϱ = −0.009; *P*_FDR = 0.984	ϱ = −0.093; *P*_FDR = 0.899	ϱ = 0.050; *P*_FDR = 0.899
Nodule dry weight	ϱ = 0.191; *P*_FDR = 0.576	ϱ = 0.019; *P*_FDR = 0.984	ϱ = 0.070; *P*_FDR = 0.899	ϱ = −0.050; *P*_FDR = 0.899
RGR	ϱ = −0.302; *P*_FDR = 0.160	ϱ = 0.222; *P*_FDR = 0.414	ϱ = 0.069; *P*_FDR = 0.899	ϱ = −0.077; *P*_FDR = 0.899

Note: ϱ, Spearman correlation coefficient; FDR, false discovery rate. No correlation remained significant after FDR correction (*P*_FDR > 0.05).
